# Diffuse alveolar hemorrhage in a patient with ANCA-associated vasculitis after thyroidectomy: A case report

**DOI:** 10.1097/MD.0000000000014630

**Published:** 2019-02-22

**Authors:** Kui-Rong Wang, Yan-Feng Zhou

**Affiliations:** Department of Anesthesiology, The First Affiliated Hospital, College of Medicine, Zhejiang University, Zhejiang, China.

**Keywords:** antineutrophil cytoplasmic antibody-associated vasculitis, case report, diffuse alveolar hemorrhage, radical thyroidectomy

## Abstract

**Rationale:**

Antineutrophil cytoplasmic antibody (ANCA)-associated vasculitis (AAV) is an autoimmune disease that mainly affects the lungs and kidneys. Limited reports of perioperative management of such patients were primarily concerned with airway stenosis. Here, we report a patient with AAV who developed diffuse alveolar hemorrhage (DAH) early after radical thyroidectomy.

**Patient concerns:**

A 57-year-old female developed wheezing and dyspnea approximately 30 minutes after radical thyroidectomy, with hemoptysis occurring the following day. The patient had a history of AAV and DAH and was maintained with prednisone.

**Diagnosis:**

A diagnosis of DAH was made on the basis of the history of AAV, dyspnea, hemoptysis, and chest computed tomography scan results that showed diffuse high-density shadows in the lungs.

**Interventions:**

The patient was administered high-dose glucocorticoids and cyclophosphamide immunosuppressive therapy. Non-invasive ventilation was needed for 2 days postoperatively due to dysfunction of oxygenation.

**Outcomes:**

After high-dose glucocorticoids and cyclophosphamide immunosuppressive therapy, DAH improved approximately 2 weeks after the surgery, during which time kidney function was not significantly impaired.

**Lessons:**

Patients with AAV may develop DAH in the early postoperative period and this may be confused with surgical complications and general anesthetic residues. Therefore, it needs to be identified in an appropriate timeframe.

## 1 Introduction

Antineutrophil cytoplasmic antibody (ANCA)-associated vasculitis (AAV) is inflammation of small blood vessels; this can have a range of clinical manifestations, from sinusitis and airway involvement only, up to fatal systemic vasculitis involving organs such as the lungs, kidneys, and peripheral nerves.^[1,2]^ Diffuse alveolar hemorrhage (DAH) is a common complication of AAV involving the lungs, and this can be life-threatening.^[1,3,4]^ Here, we report a case of DAH that developed after radical thyroidectomy in a patient with AAV.

## 2 Case report

Approval was obtained from the Ethics Committee of the First Affiliated Hospital, College of Medicine, Zhejiang University for reporting of this case.

**Figure 1:**
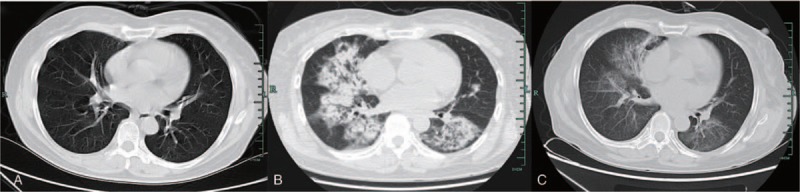
Chest computed tomography scan findings of the patient. A. One month before surgery, multiple fibro proliferative foci were seen in the lungs. B. Two days after surgery, multiple plaque-like high-density shadows were seen in both lungs, and the margins were unclear. C. Two weeks postoperatively, patchy high-density shadows were significantly absorbed compared to that of 2 days after surgery.

A 57-year-old female was diagnosed with thyroid cancer following a biopsy and underwent a radical thyroidectomy. Two years prior, the patient had been diagnosed with AAV complicated with DAH after developing hemoptysis, anemia, and an increased erythrocyte sedimentation rate. At that time, a large dose of methylprednisolone (500 mg/day intravenously for 3 days) was administered. The intravenous infusion of methylprednisolone was then gradually reduced. Her condition gradually improved approximately 2 weeks after treatment initiation, and her serum creatinine remained within the normal range (50–60 μmol/L (0.57–0.68 mg/L)). Following this, the patient was prescribed oral prednisone, with the dose gradually reduced to 10 mg/day for maintenance. One month before surgery, she developed fatigue, hematuria, and proteinuria, and her serum creatinine was 278 μmol/L (3.1 mg/dL). She was diagnosed with renal lesions caused by AAV. Lung fibrosis was noted on a computed tomography (CT) scan performed at that time (Fig. 1A). High-dose methylprednisolone was administered again (500 mg/day intravenously for 3 days and then gradually reduced), and a total of 1.0 g of cyclophosphamide was also intravenously infused twice (0.5 g at a time). Following this, the patient was prescribed oral prednisone, with the dose gradually reduced. The prednisone dose was 35 mg/day before surgery.

Although serum perinuclear ANCA was only positive once in the month before the surgery in this case, and serum cytoplasmic ANCA was not detected, the diagnosis of AAV was pathologically confirmed by renal biopsy about 1 month before surgery. In this time period, there were 2 instances where the anti-myeloperoxidase antibody was 2 times higher than the upper limit of detection (2 years before surgery and 1 month before surgery), whereas the anti-protease 3 antibody remained within the normal range. Enzyme-linked immunosorbent assay was used to detect all above-mentioned antibodies.

In the comprehensive examination performed owing to impaired renal function 1 month before surgery, B-ultrasonography revealed a thyroid mass. Based on the biopsy results, the patient was diagnosed with papillary thyroid cancer; therefore, surgery was performed. The patient had no hemoptysis or shortness of breath before the operation and there was no abnormality noted during lung auscultation. Chest X-ray examination found no abnormality. The serum creatinine level was 216 μmol/L (2.4 mg/dL).

On the day of surgery, the prednisone dose was administered, and 40 mg methylprednisolone was injected intravenously before induction of anesthesia. The ID 7.0 mm tracheal tube was inserted under general anesthesia, and radical thyroidectomy was successfully completed in approximately 2 hours. The patient regained consciousness at the completion of the operation and the endotracheal tube was removed immediately.

The patient developed dyspnea and wheezing 30 minutes after tracheal extubation. In order to exclude an injury of the recurrent laryngeal nerve from thyroid surgery, a video laryngoscopy was performed. This showed normal vocal cord activity; however, for the prevention of laryngeal edema, methylprednisolone 40 mg was administered intravenously. Auscultated lung sounds were loud, and the patient was sent to the intensive care unit for further observation and treatment.

On the first day after surgery, hemoptysis occurred with dyspnea. Large-flow oxygen inhalation was required to maintain an oxygen saturation of approximately 90%, and non-invasive ventilation was required for 2 days postoperatively. Dyspnea gradually improved 2 days after surgery with administration of methylprednisolone (240 mg/day), atomization inhalation, and hemostasis treatment. Hemoptysis gradually reduced 5 days after surgery. Five days after the surgery, methylprednisolone was increased to 500 mg intravenously every day for 3 days, and a total of 1 g of cyclophosphamide was administered intravenously twice (0.5 g at a time). The hemoptysis disappeared approximately 2 weeks after surgery.

**Figure 2:**
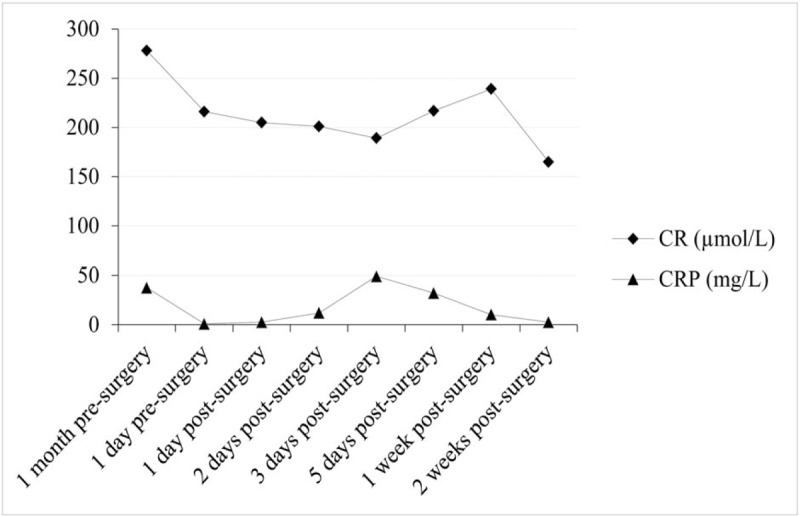
Changes in serum CR and CRP during the surgery. CR = creatinine, CRP = C-reactive protein.

The CT scans from 2 days and 2 weeks postoperatively are shown in Figure 1. AAV was diagnosed based on the patient's medical history, clinical symptoms, and the CT examination performed 2 days postoperatively, which found high-density shadows in the lungs (Fig. 1B). High-density shadows were less evident in the CT scan 2 weeks after surgery (Fig. 1C). In the CT examination from 2 days after surgery, 3-dimensional reconstruction of the airway was performed to rule out dyspnea caused by an abnormal airway. No abnormality was found. Fortunately, during this postoperative episode of DAH, no further impairment of renal function was observed, as no significant change in urine output or serum creatinine occurred. Serum creatinine changes are shown in Figure 2.

## 3 Discussion

Although immunosuppressive therapy, such as glucocorticoids and cyclophosphamide, has significantly improved the survival rate of AAV from a 2-year mortality rate of 90% to a 2-year survival rate of more than 80%,^[1]^ morbidity and mortality rates remain high, as life-threatening complications such as renal impairment, DAH, cerebral lesions, and cardiovascular events often occur.^[5]^ Neutrophils are the main driver of tissue damage in AAV, and complement C5a receptor 1 (C5aR1) plays a crucial role in the activation of the complement pathway in AAV.^[6]^ Therapeutic methods mainly include high-dose glucocorticoids and other immunosuppressive drugs, such as cyclophosphamide. A low-dose glucocorticoid is usually maintained during the stabilization phase.^[2,7]^

Reports on the perioperative management of AAV patients are limited. Previous reports include a patient with aortic aneurysm with AAV who successfully underwent surgery,^[8]^ and a patient with AAV due to subglottic stenosis, misdiagnosed as asthma before surgery, that led to a tracheotomy because tracheal intubation was difficult during surgery.^[9]^

In our case, the symptoms of dyspnea occurred in a short period of time after radical thyroidectomy. Therefore, the complications of radical thyroidectomy, such as recurrent laryngeal nerve injury, were first considered,^[10]^ and video laryngoscopy was performed to exclude vocal cord paralysis. As dyspnea occurred during recovery from general anesthesia, residual anesthetic, and analgesic medications needed to be considered and ruled out.^[11]^ According to the patient's previous medical history and hemoptysis, DAH was diagnosed on the second day after surgery, and CT scan confirmed this diagnosis. To rule out comorbid airway lesions, airway 3-dimensional reconstruction was performed and no abnormalities were found. The inner diameter of a 7 mm tracheal tube was inserted smoothly during the operation. Therefore, combined airway stenosis was ruled out. Fortunately, in this case, DAH was treated effectively with high-dose glucocorticoids and cyclophosphamide. It has been reported that DAH may be associated with severe and persistent hypoxemia and might even require extracorporeal membrane oxygenation; these cases have a high mortality rate.^[4,12]^

This patient developed DAH soon after surgery. As the patient was on glucocorticoid replacement therapy, glucocorticoid was routinely administered before surgery. However, as a complication of AAV, DAH can occur within a short period of time after surgery. Perioperative use of equivalent maintenance doses or stress doses of glucocorticoids (equivalent to 4 times the therapeutic dose) in patients who require glucocorticoid maintenance as immunosuppressive therapy is controversial. However, the primary concern of patients is that adrenal cortical atrophy may occur after long-term, high-dose glucocorticoid treatment, and insufficient glucocorticoid secretion under stress may cause refractory hypotension as a manifestation of “cortical crisis.”^[13,14]^ At present, there are no guidelines for the use of high-dose glucocorticoids or even other immunosuppressive agents to prevent AAV complications during the perioperative period.^[2,5]^

In conclusion, this case report shows that AAV patients may develop DAH postoperatively, which may occur early in the operation and may be confused with surgical complications and general anesthesia residues, highlighting the need for it to be identified in an appropriate timeframe.

## Author contributions

**Data curation:** Kui-Rong Wang.

**Writing – original draft:** Yan-Feng Zhou.

**Writing – review & editing:** Kui-Rong Wang, Yan-Feng Zhou.
